# Molecular Characterization and Clinical Relevance of ALDH2 in Human Cancers

**DOI:** 10.3389/fmed.2021.832605

**Published:** 2022-01-13

**Authors:** Bo Ma, Zaoqu Liu, Hui Xu, Long Liu, Tao Huang, Lingfang Meng, Libo Wang, Yuyuan Zhang, Lifeng Li, Xinwei Han

**Affiliations:** ^1^Department of Interventional Radiology, The First Affiliated Hospital of Zhengzhou University, Zhengzhou, China; ^2^Interventional Institute of Zhengzhou University, Zhengzhou, China; ^3^Interventional Treatment and Clinical Research Center of Henan Provice, Zhengzhou, China; ^4^Department of Hepatobiliary and Pancreatic Surgery, The First Affiliated Hospital of Zhengzhou University, Zhengzhou, China; ^5^Medical School, Huanghe Science and Technology University, Zhengzhou, China; ^6^Department of Infection Management, The Second Affiliated Hospital of Zhengzhou University, Zhengzhou, China; ^7^Internet Medical and System Applications of National Engineering Laboratory, Zhengzhou, China

**Keywords:** ALDH2, pan-cancer, genetic alterations, drug sensitivity, immunotherapy, survival

## Abstract

**Background:** Aldehyde dehydrogenase 2 (ALDH2) is well-known to be a key enzyme in alcohol metabolism. However, a comprehensive understanding of ALDH2 across human cancers is lacking.

**Methods:** A systematic and comprehensive analysis of the molecular alterations and clinical relevance for ALDH2 in more than 10,000 samples from 33 cancer types was performed. qRT-PCR was performed on 60 cancer and 60 paired nontumor tissues.

**Results:** It was observed that ALDH2 was generally downregulated in most cancers, which was mainly driven by DNA hypermethylation rather than mutations or copy number variations. Besides, ALDH2 was closely related to the inhibition and activation of tumor pathways and a variety of potential targeted agents had been discovered in our research. Last but not least, ALDH2 had the best prediction efficacy in assessing immunotherapeutic response compared with PD-L1, PD-1, CTLA4, CD8, and tumor mutation burden (TMB) in cutaneous melanoma. According to the analysis of large-scale public data and 60 pairs of clinical cancer samples, we found the downregulation of ALDH2 expression tends to suggest the malignant phenotypes and adverse prognosis, which might enhance the precise diagnosis and timely intervention of cancer patients.

**Conclusion:** This study advanced the understanding of ALDH2 across cancers, and provided important insight into chemotherapy, immunotherapy and prognosis of patients with cancer.

## Introduction

Aldehyde dehydrogenase 2 (ALDH2) is one of the aldehyde dehydrogenase family of enzymes. As another member of the aldehyde dehydrogenase family, ALDH1 is widely known for its key role in carcinogenesis and cancer treatment (1). Nonetheless, ALDH2 is well-known to be a key enzyme in alcohol metabolism and has a major contribution in oxidizing endogenous aldehydic products arising from lipid peroxidation such as 4-hydroxy-2-nonenal (4-HNE) and malondialdehyde under oxidative stress (2). The dysfunction of ALDH2 has a tight correlation with multiple diseases such as diabetes, cardiovascular disease, osteoporosis, and cancers (3). As the rate-limiting enzyme of alcohol metabolism, ALDH2 has a profound impact on the clearance process of toxic acetaldehyde. Low expression or genetic polymorphism of ALDH2 dramatically reduce its enzyme activity, and the accumulation of acetaldehyde can further damage normal cells and lead to cancers (4).

Previous studies have shown that ALDH2 has profound impacts on the prognosis and treatment of tumors. For example, prognostic analysis revealed the low expression of ALDH2 indicated adverse overall survival (OS) in patients with lung, liver, or bladder cancer (5, 6). Hou et al. have demonstrated that ALDH2 modulates the adenosine 5 '-monophosphate (AMP) -activated protein kinase pathway in mice, which in turn inhibits the progression of hepatocellular carcinoma (7). ALDH2 has also been reported to regulate tumor proliferation by catabolizing some endogenous substrates derived from normal cell metabolism (e.g., 4-HNE) (8). In addition, ALDH2 has been demonstrated to be tightly associated with the sensitivity and resistance of multiple targeted drugs including 4-Hydroxycyclophosphamide, Doxorubicin, Cisplatin, Vincristine, and Disulfiram (9–12). Therefore, a comprehensive understanding of ALDH2 on the molecular characterization and clinical relevance across human cancers is necessary. Understanding the abnormal expression and genomic alterations of ALDH2 may help elucidate its role in cancer prognosis and therapy.

In this study, we are committed to systematically and comprehensively exploring the molecular alterations, prognosis, and therapeutic value of ALDH2 in 33 cancer types. It was proven that aberrant expression and genetic alterations of ALDH2 are widespread in different types of cancer. By assessing the correlation between ALDH2 and cancer pathway activity as well as its clinical value, ALDH2 was found to be a potential biomarker for immunotherapeutic evaluation and prognostic stratification. In a word, our study demonstrates the important value of ALDH2 in cancer and lays the foundation for translational medicine development of ALDH2.

## Materials and Methods

### Multi-Omics Data of 33 Cancer Types in TCGA

33 different cancer cohorts were collected from The Cancer Genome Atlas Research Network (TCGA, https://portal.gdc.cancer.gov/). The RNA-seq, copy number variation (CNV), and survival information were downloaded from UCSC Xena website (https://xenabrowser.net/datapages/). The somatic mutation (VarScan2 variant aggregation and masking) and HumanMethylation450 array were derived from TCGA GDC portal. Tumor mutation burden (TMB) was defined as the total number of non-silent somatic mutations in each sample (13). The correlation between methylation sites and ALDH2 expression was evaluated by Pearson Correlation Coefficient and the methylation site with r < −0.3 and false discovery rate (FDR) <0.05 was considered as hypermethylation. The proteomics data were retrieved from the Clinical Proteomic Tumor Analysis Consortium (CPTAC, https://proteomics.cancer.gov/programs/cptac), and the protein expression levels of ALDH2 in human tumors and normal tissues were determined by Human Protein Atlas (HPA, https://www.proteinatlas.org/). See Supplementary Material for the detailed description.

### Immunotherapeutic Cohorts

Three independent cohorts containing immunotherapy information and expression data were retrieved from Gene Expression Omnibus (GEO, https://www.ncbi.nlm.nih.gov/geo/). (1) GSE100797: melanoma patients treated with adoptive T cell therapy (ACT) (14); (2) GSE78220: melanoma patients treated with anti-PD-1 (15); (3) GSE91061: melanoma patients treated with anti-PD-1 (16). Based on the RECIST v1.1 criterion, 21 patients in GSE100797, 28 patients in GSE78220, as well as 49 patients in GSE91061 were finally determined. A detailed description is provided in the Supplementary Material.

### Estimation of Drug Response

Predicted responses of cancer patients to 138 anticancer drugs were collected from existing studies to evaluate drug response for TCGA samples (17). Half maximum inhibitory concentration (IC50) was used to assess drug sensitivity. Besides, the correlation between imputed drug response and ALDH2 mRNA expression was calculated by Pearson correlation and drugs with top 10 |*r*| and FDR <0.05 were considered to be the latent targets for ALDH2.

### Oncogenic Pathway Activity and Immune Infiltration Assessment

The FPKM normalized gene expression of TCGA database was performed to Gene Set Variation Analysis (GSVA), which is a non-parametric, unsupervised method for estimating variation of gene set enrichment through the samples of an expression data set. The correlation between ALDH2 and oncogenic pathways was verified by Gene set enrichment analysis (GSEA). 50 gene sets were enrolled from Molecular Signatures Database (MSigDB, https://www.gsea-msigdb.org/gsea/msigdb/) v7.2 Hallmark database and FDR <0.05 was considered statistically significant. Single sample gene set enrichment analysis (ssGSEA) algorithm implemented in GSVA package was applied to estimate the relative infiltration abundance of tumor microenvironment (TME) cells. The gene sets for marking 28 immune cell types were collected from Charoentong et al. (18). Furthermore, we evaluated the correlation between ALDH2 expression and pathway or cell activity using Pearson correlation coefficient.

### Human Cancer Specimens and Clinical Information

A total of 60 cancer tissues and matched adjacent nontumor tissues were enrolled from The First Affiliated Hospital of Zhengzhou University, including 5 pairs of pancreatic cancers, 10 pairs of paired liver cancers, 5 pairs of bile duct cancers, and 40 pairs of colorectal cancers. All patients signed written informed consent. See Supplementary Material for the inclusion criteria of patients. The clinical characteristics of patients included gender, clinical stage, distant metastasis status, lymph metastasis status, vessel invasion status, nerve invasion status, disease-free survival (DFS), and OS. Refer to Supplementary Table S1 for details of the baseline information.

### RNA Preparation and Quantitative Real-Time PCR

Total RNA was isolated from cancer tissues and paired adjacent nontumor tissues with RNAiso Plus reagent (Takara, Dalian, China) according to the manufacturer's instructions. RNA quality was evaluated using a NanoDrop One C (Waltham, MA, USA), and RNA integrity was assessed using agarose gel electrophoresis. An aliquot of 1 μg of total RNA was reverse-transcribed into complementary DNA (cDNA) according to the manufacturer's protocol using a High-capacity cDNA Reverse Transcription kit (TaKaRa BIO, Japan). Quantitative real-time PCR (qRT-PCR) was performed using SYBR Assay I Low ROX (Eurogentec, USA) and SYBR® Green PCR Master Mix (Yeason, Shanghai, China) to detect the expression. The data were normalized to the expression of GAPDH. The sequences of the primers were as follows:

GAPDH forward (5'- to 3'-): GGAGCGAGATCCCTCCAAAAT

GAPDH reverse (5'- to 3'-): GGCTGTTGTCATACTTCTCATGG

ALDH2 forward (5'- to 3'-): GTTTGGAGCCCAGTAACCCTT

ALDH2 reverse (5'- to 3'-): CCCACACTCACAGTTTTGAATT.

### Statistical Analysis

The correlation between two variables was assessed using Pearson correlation. Correlations with | r | > 0.3 and FDR < 0.05 were significant and labeled as “SigCor” in the genomic analysis. The *survminer* package was utilized to determine the optimal cut-off value of ALDH2 expression based on survival information. The *survival* package was employed for Kaplan-Meier survival analysis and the different significance was defined by the log-rank test. The *pROC* package was utilized to plot the receiver operating characteristic (ROC) curves. The area under ROC curve (AUC) was used to compare the accuracy of predicting immunotherapeutic response of ALDH2, CD8, CTLA-4, PD-1, PD-L1, and TMB. Differences in ALDH2 expression between the two groups were compared by Wilcoxon rank sum test or independent samples *T*-test. Multiple comparisons were performed using ANOVA or Kruskal-Wallis tests. Paired *T*-test was used to analyze ALDH2 expression differences between paired tumor samples and matched adjacent nontumor samples. All statistical *P* values were two-sided, and *P* < 0.05 was defined as statistically significant. Adjust *P* value was performed using Benjamini-Hochberg (BH) multiple test correction. All plotting and data processing were completed in R 4.0.2 software.

## Results

### Multi-Omics Landscape of ALDH2 in Cancers

**Figure 1:**
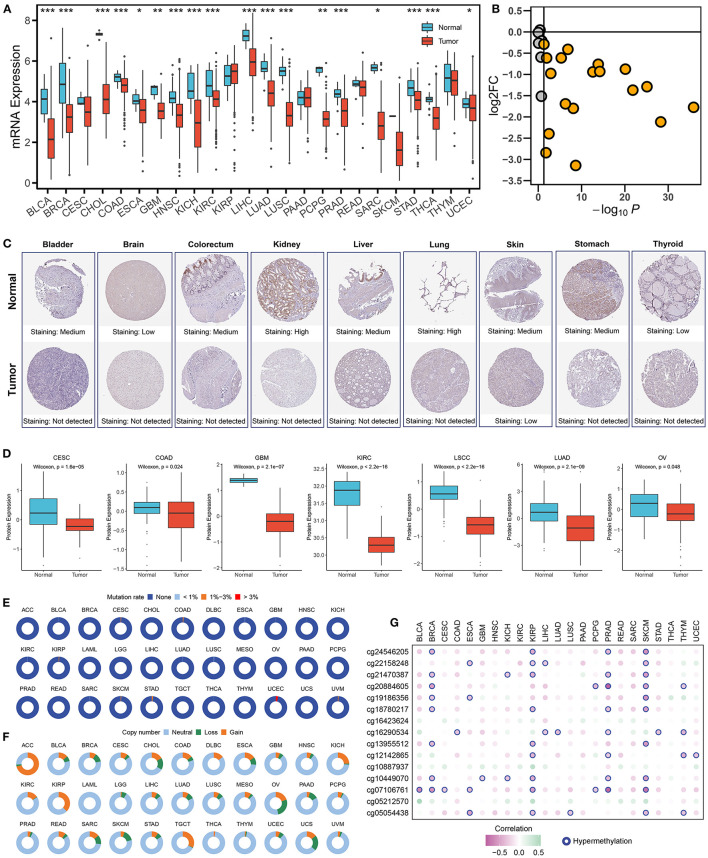
Multi-omics landscape of ALDH2 in cancers. **(A)** The expression of ALDH2 between normal and tumor tissue across human cancers. Wilcoxon rank-sum test: *P < 0.05; **P < 0.01; ***P < 0.001. **(B)** The log2 fold change (FC) and *P*-value distribution of ALDH2 between normal and tumor across human cancers. **(C)** The immunohistochemical results displayed that lighter protein staining in tumor relative to normal tissue. **(D)** The protein level of ALDH2 between normal and tumor across human cancers. **(E)** The mutation frequency of ALDH2 across 33 cancer types. **(F)** The copy number variation frequency of ALDH2 across 33 cancer types. **(G)** Pearson correlation between the expression of ALDH2 and the methylation beta value of 15 corresponding CpG site. Hypermethylation represents the methylation site with *r* < −0.3 and FDR < 0.05.

As illustrated in Figures 1A,B, ALDH2 presented universally lower mRNA expression in almost all tumors. Immunohistochemical results also displayed that lighter protein staining in tumors relative to normal tissue (Figure 1C). The proteomics data of multiple cancers further validated these results in protein level (Figure 1D). The above suggested ALDH2 was significantly downregulated in human cancers. In order to clarify the factors affecting ALDH2 expression, we further investigated the genomic variation of ALDH2 across tumors. Interestingly, despite ALDH2 polymorphism being closely related to many diseases such as cardiovascular and digestive diseases, the mutational events were extremely rare in cancers, and the mutation rate of most cancers were <1% (Figure 1E). Therefore, the mutation is not the major driver for the downregulation of ALDH2. Conversely, ALDH2 presented extensive copy number variation (CNV) in cancers (Figure 1F). To assess the impact of CNV on ALDH2 expression, we further investigated the correlation between CNV and expression of ALDH2. Notably, CNV had little impact on ALDH2 expression in most cancers, which also indicated CNV was not an important factor downregulating ALDH2 expression (Supplementary Figure S1). DNA hypermethylation is another essential factor that regulates gene expression, which is also a ubiquitous feature of carcinogenesis. ALDH2 has 15 methylation sites in HumanMethylation450 array. Our results indicate that the expression of ALDH2 in most tumors was negatively correlated with DNA methylation. The multiple cancers displayed plenty of hypermethylation sites, particularly KIRP and SKCM (Figure 1G). Overall, the downregulation of ALDH2 was driven by DNA hypermethylation rather than mutation or CNV.

### Functional Analysis of ALDH2

**Figure 2:**
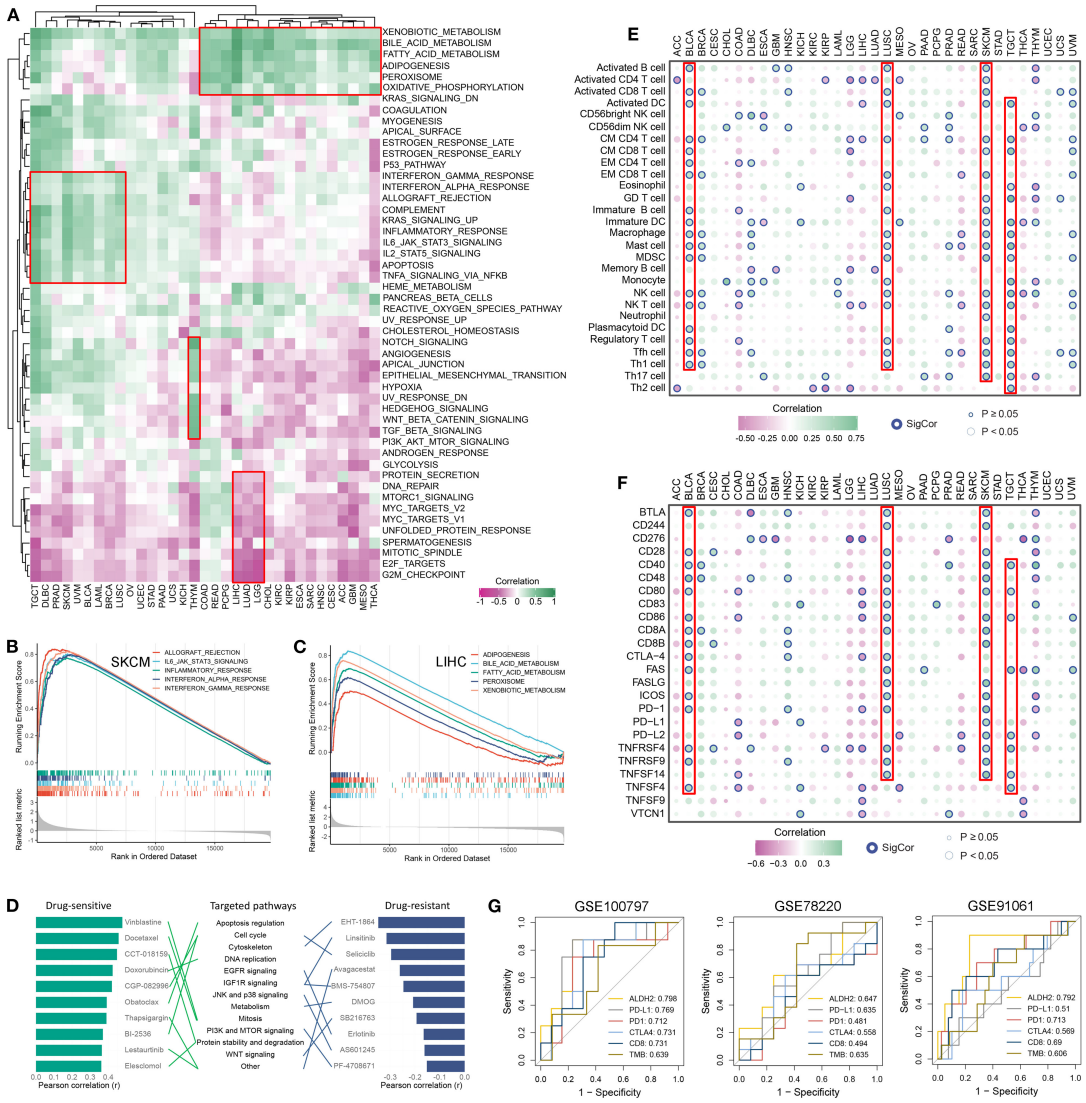
Functional analysis and cancer therapy implication of ALDH2. **(A)** Correlation between ALDH2 expression and 50 oncogenic pathways activity across 33 cancer types. **(B,C)** The top 5 pathways with FDR <0.05 and NES >0 was retrieved form GSEA results of SKCM **(B)** and LIHC **(C)**. **(D)** The potential roles of ALDH2 in cancer drug therapy. **(E,F)** Correlation between ALDH2 expression and 28 immune cells infiltration **(E)** and 24 immune checkpoints expression **(F)** across 33 cancer types. **(G)** Comparison of the predictive accuracy of ALDH2, PD-L1, PD-1, CTLA4, CD8, and TMB for immunotherapy in GSE100797, GSE78220, and GE91061.

The correlation between ALDH2 expression and the activity of pathways involved in 50 cancer hallmarks was explored to further understand the molecular mechanism of ALDH2 involvement in cancer. The results indicate that ALDH2 expression was closely correlated with inhibition or activation of multiple oncogenic pathways (Figure 2A). For instance, the expression of ALDH2 was predominantly positive related to immune related pathways such as interferon and complement response in TGCT, DLBC, and SKCM, metastasis-related pathways such as epithelial mesenchymal transition and angiogenesis in THYM, and lipid metabolism-related pathways such as adipogenesis and fatty acid metabolism in COAD, READ, and LIHC, etc. Moreover, ALDH2 expression also had a significantly negative correlation with proliferation-related pathways such as DNA repair, MYC and E2F targets, and G2M checkpoint in LIHC, LUAD, and LGG, etc. Based on the ALDH2 expression group, GSEA analysis verified the above results. SKCM presented intense immune-related pathways (Figure 2B); LIHC significantly enriched in lipid metabolism-related pathways (Figure 2C); THYM displayed canonical metastasis-related pathways (Supplementary Figure S2A); and LUAD was negatively related to proliferation-related pathways (Supplementary Figure S2B). Collectively, ALDH2 might play multiple functional roles in distinct cancers.

### Implications of ALDH2 for Cancer Chemotherapy and Immunotherapy

Furthermore, based on the relationship between ALDH2 expression and impute drug response of each patient, we identified the potential roles of ALDH2 in cancer drug therapy (Figure 2D). Obviously, we found ALDH2 might enhance the drug sensitivity of Vinblastine, Docetaxel, CCT-018159, Doxorubicin, CGP-082996, Obatoclax, Thapsigargin, BI-2536, Lestaurtinib, and Elesclomol; elevate the drug resistant of EHT-1864, Linsitinib, Selicicib, Avagacestat, BMS-754807, DMOG, SB216763, Erlotinib, AS601245, and PF-4708671. Some of our results are consistent with previous studies (6–8). Of note, ALDH2 may have different drug responses to the same targeted pathway. For example, the targeted pathway of Obatoclax, BI-2536, and Selicicib is cell cycle, but ALDH2 enhances drug sensitivity of Obatoclax and BI-2536, and decreases the drug sensitivity of Selicicib. These results might advance clinical drug selection and facilitate precision therapy in human cancers.

Due to the enriched immune-related pathway across cancers (Figures 2A,B), we further assessed the correlation between ALDH2 expression and 24 immune checkpoints expression and 28 immune cells infiltration (Figures 2E,F). It was observed that predominantly correlation in multiple cancers, particularly in those with well immunotherapeutic efficacy, such as BLCA, LUSC, SKCM, and TGCT. Thus, we hypothesized ALDH2 might be a latent biomarker for predicting immunotherapeutic response. Three independent SKCM cohorts containing expression data and immunotherapeutic information were enrolled. We compared the accuracy of ALDH2 in predicting immunotherapeutic response with that of existing biomarkers such as CD8, CTLA4, PD-1, PD-L1, and TMB. The results demonstrated that ALDH2 had the highest accuracy in predicting the effect of immunotherapy compared with other biomarkers (Figure 2G). These findings suggested ALDH2 was a promising biomarker for assessing immunotherapeutic efficacy in SKCM.

### Clinical Relevance of ALDH2 Across Cancers

**Figure 3:**
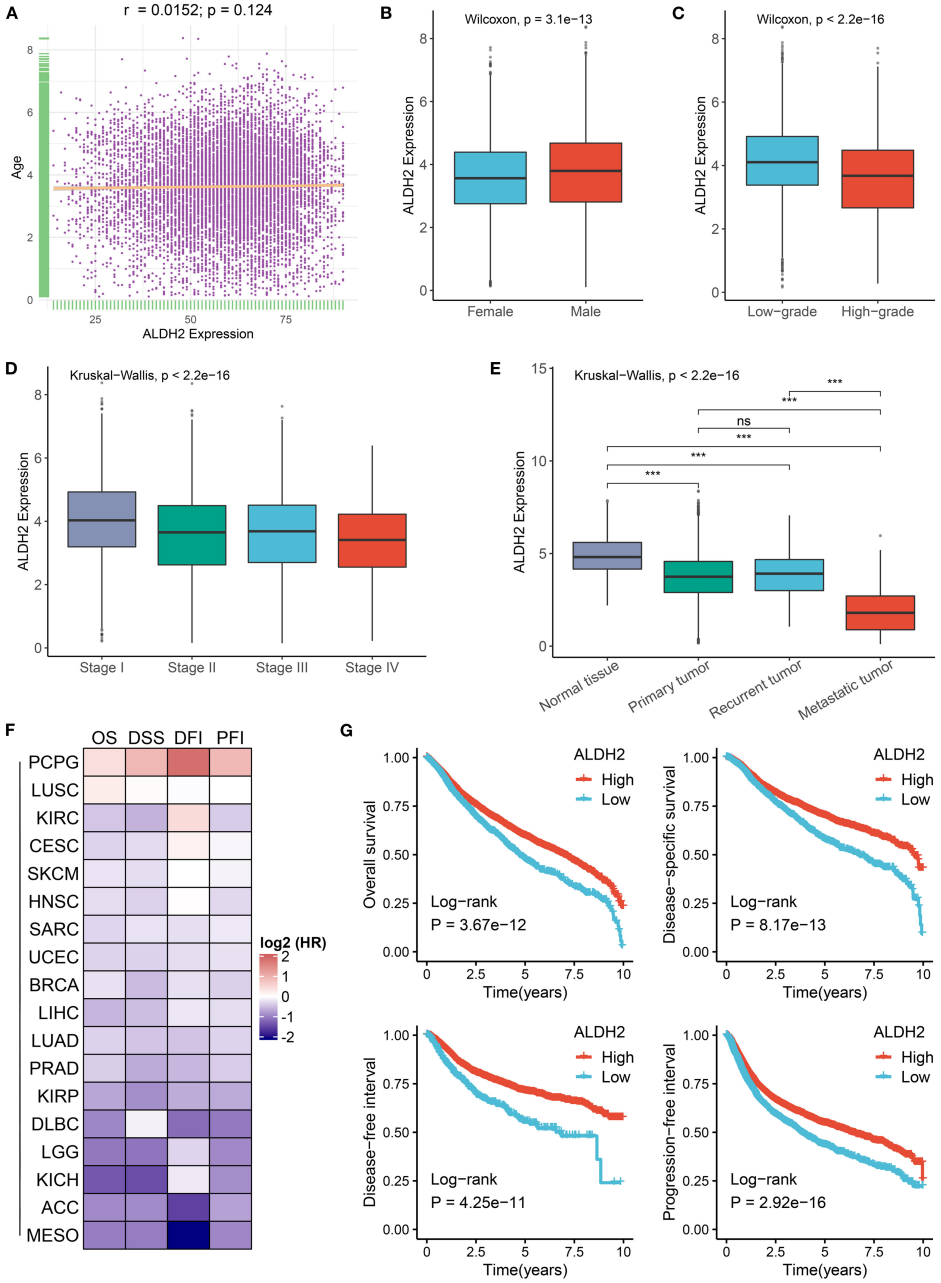
Clinical relevance of ALDH2 across cancers. **(A)** Correlation between ALDH2 expression and age in cancers. **(B)** The difference of ALDH2 expression between female and male groups. **(C)** The difference of ALDH2 expression between low grade (G1, G2 or low grade in cancers) and high grade (G3, G4 or high grade in cancers). **(D)** The distribution of ALDH2 expression among distinct AJCC stages. **(E)** The distribution of ALDH2 expression among different categories tissues including solid normal tissue, primary tumor, recurrent tumor, and metastatic tumor. ns, *P* > 0.05; ***P < 0.001. **(F)** Heatmap showing the hazard ratio (HR) of overall survival, disease specific survival, disease free interval, and progression free interval for ALDH2 in human cancers. The cell value represents log2 (HR). **(G)** Kaplan-Meier survival curve of ALDH2 for overall survival, disease specific survival, disease free interval, and progression free interval.

We further revealed the clinical value of ALDH2 in cancers subsequently. As shown in our results, there was no correlation between ALDH2 expression and age (Figure 3A). Male patients had predominant higher ALDH2 expression relative to female patients (Figure 3B). In addition, downregulation of ALDH2 was associated with malignant phenotypes of cancers (Figures 3C,D). Notably, metastasis tumors had the lowest ALDH2 expression compared with primary and recurrent tumors, and there was no significant difference between recurrent and primary tumors (Figure 3E). Furthermore, univariate Cox regression revealed ALDH2 was a protective factor of survival (disease-specific survival, overall survival, progression-free interval, and disease-free interval) in most cancers (Figure 3F). The Kaplan-Meier survival analysis also demonstrated the same results in human cancers (Figure 3G). The significant clinical relevance of ALDH2 may provide important insight into translational medicine developments.

### Validating the Role of ALDH2 Across Tumors in Our Cohort

**Figure 4:**
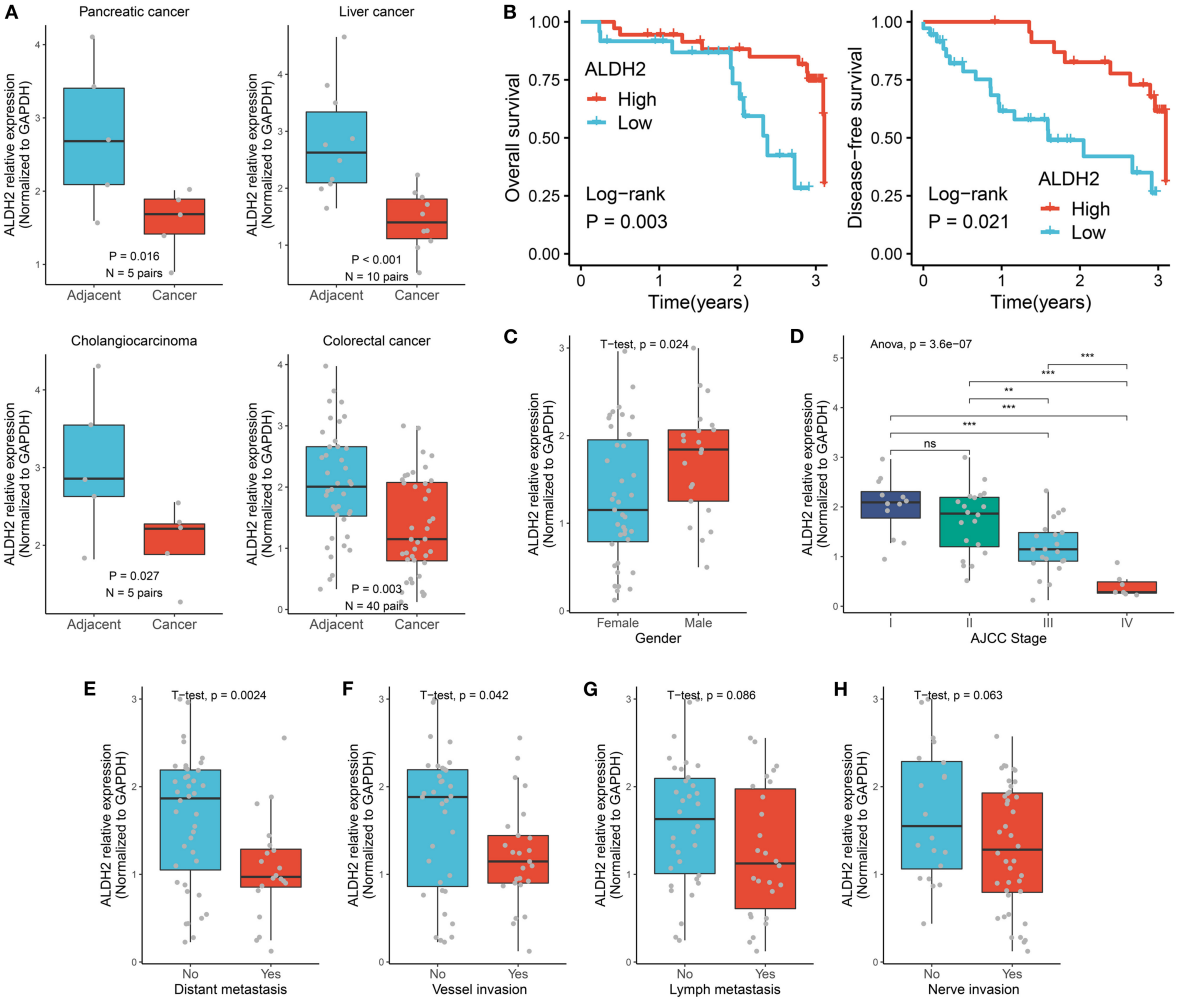
Validating the role of ALDH2 across tumors in our cohort. **(A)** qRT-PCR displayed the mRNA expression level of ALDH2 between cancer tissues and their corresponding adjacent nontumor tissues. **(B)** Kaplan-Meier survival curve of ALDH2 for overall survival, disease-free survival. **(C)** The difference of ALDH2 expression between female and male groups. **(D)** The distribution of ALDH2 expression among distinct AJCC stages. *T*-test: ns, *P* > 0.05; **P < 0.01; ***P < 0.001. **(E)** The difference of ALDH2 expression in the groups with or without distant metastasis. **(F)** The difference of ALDH2 expression in the groups with or without vessel invasion. **(G)** The difference of ALDH2 expression in the groups with or without lymph metastasis. **(H)** The difference of ALDH2 expression in the groups with or without nerve invasion.

A total of 60 cancer tissues and matched adjacent nontumor tissues were enrolled from The First Affiliated Hospital of Zhengzhou University, including 5 pairs of pancreatic cancers, 10 pairs of paired liver cancers, 5 pairs of bile duct cancers, and 40 pairs of colorectal cancers. As illustrated in Figure 4A, the expression of ALDH2 was significantly downregulated across all cancers relative to their corresponding adjacent nontumor tissues (*T*-test: all *P* < 0.05). Patients with high ALDH2 expression possessed better OS and DFS compared to patients with low ALDH2 expression (Log-rank test: both *P* < 0.05; Figure 4B). Moreover, highly expressed ALDH2 was more likely to appear in male tumor patients (*T*-test: *P* < 0.05; Figure 4C). Consistent with the above results, the low expression of ALDH2 tended to indicate a malignant clinical outcome. For example, patients with malignant phenotypes such as advanced AJCC stage, distant metastasis, and vessel invasion had the inferior ALDH2 expression (All *P* < 0.05; Figures 4D–F). Of note, when lymph metastasis or nerve invasion events occurred, patients had predominantly downregulated ALDH2 expression although there were no statistically significant (Figures 4G,H). Overall, the downregulation of ALDH2 expression tends to suggest malignant phenotypes, which might enhance the precise diagnosis and timely intervention of cancer patients.

## Discussion

In our study, we systematically and comprehensively explored the molecular alterations, prognosis, and therapeutic value of ALDH2 in 33 cancer types. It was found that ALDH2 was downregulated in most cancer, which was mainly driven by DNA hypermethylation rather than mutation or CNV. The aberrant expression of ALDH2 is dramatically correlated with the activity of cancer pathways. Moreover, ALDH2 was proved to be a potentially useful biomarker for immunotherapy assessment and prognostic stratification.

ALDH2 is a mitochondrial enzyme that is closely associated with the degradation of acetaldehyde. Acetaldehyde is a toxic substance, and its accumulation in cells causes acetaldehyde-induced DNA interstrand crosslinks (AA-ICLs), which further induces the initiation and progression of many tumors such as liver cancer, colorectal cancer, gastric cancer, esophageal cancer, and lung cancer (4, 19). In this study, we demonstrated that ALDH2 is defective in most tumors relative to normal tissues on both mRNA and protein expression levels, which was further validated by *in vitro* experiments. In order to find out the potential reasons leading to the downregulation of A LDH2 expression. We analyzed somatic mutations, CNV, and DNA methylation of ALDH2 across 33 cancer types. We observed that ALDH2 displayed low mutations. Previous researches demonstrated ALDH2 polymorphism was common in the Asian population (4, 19). As far as we know, the population of TCGA was mainly from the United States. Thus, the aberrant expression of ALDH2 was not driven by mutation in the United States. Notably, ALDH2 displayed broad CNV alterations, while there was no significant correlation between the expression and CNV. Finally, we found that ALDH2 hypermethylation was an essential mechanism in the downregulation of ALDH2 in human cancers.

Our research demonstrated the close correlation between the aberrant expression of ALDH2 and the activity of cancer pathways. Of note, ALDH2 might be involved in the different processes of initiation and progression in different tumors. For instance, ALDH2 is mainly associated with metabolic pathways in respiratory and digestive system tumors, metastasis-related pathways in THYM, and immune-related pathways in SKCM. The multiple roles of ALDH2 in cancers might give rise to diverse reflections on cancer treatments. Previous studies have proved that ALDH2 is closely correlated with the sensitivity and resistance of multiple targeted drugs such as 4-Hydroxycyclophosphamide, Doxorubicin, Cisplatin, Vincristine, and Disulfiram (9–12). In this study, based on the imputed tumor response of 138 anticancer-drugs from a large cohort, we provided potential sensitive or resistant drug resources for ALDH2-deficient tumors. Moreover, due to the enriched immune-related pathway and immune infiltration in SKCM, we further assessed the accuracy of ALDH2 in predicting immunotherapy response of three SKCM cohorts, and found the predictive performance of ALDH2 was better than popular indicators such as CD8, CTLA4, PD-1, PD-L1, and TMB. This interesting finding suggested that ALDH2 might be a reliable and promising biomarker for evaluating immunotherapy response in SKCM.

In addition, we have proven ALDH2 has significant clinical value in both large-scale public datasets and 60 pairs of clinical cancer samples. We found that male patients have a superior expression of ALDH2 compared with female patients. Patients with low ALDH2 expression possessed an adverse prognosis. The low expression of ALDH2 tended to indicate malignant phenotypes such as advance stage, high metastasis tendency, and vessel invasion. These results indicated ALDH2 deficiency is a good indicator for assessing the clinical outcome of patients with cancer.

Collectively, we have demonstrated ALDH2 is prevalent downregulation in most cancers, which is mainly driven by DNA hypermethylation rather than mutation or CNV. ALDH2 is not only tightly associated with the activation and inhibition of cancer pathways, but also displays a predominant correlation with the sensitivity and resistance of multiple drugs, and has the best prediction efficacy in assessing immunotherapeutic response compared with CD8, CTLA4, PD-1, PD-L1, and TMB in SCKM. In addition, the deficiency of ALDH2 tends to suggest malignant phenotypes and adverse prognosis, which might enhance the precise diagnosis and timely intervention of cancer patients. This study advanced the understanding of ALDH2 across cancers, and laid a foundation for the translational medicine developments of ALDH2.

## Data Availability Statement

The datasets presented in this study can be found in online repositories. The names of the repository/repositories and accession number(s) can be found in the article/Supplementary Material.

## Ethics Statement

The studies involving human participants were reviewed and approved by Ethics Committee of the First Affiliated Hospital of Zhengzhou University. The patients/participants provided their written informed consent to participate in this study.

## Author Contributions

ZL made the conceptualization, data curation, formal analysis, investigation, methodology, resources, software, validation, visualization, and writing. BM supported the funds and reviewed the manuscript. HX, LoL, TH, LM, LW, YZ, LiL, and XH reviewed and edited the manuscript. All authors agree to publish this manuscript.

## Funding

This study was supported by Key Research Projects of Henan Higher Education (No.16A320053) and Youth Innovation Fund of the First Affiliated Hospital of Zhengzhou University to BM.

## Conflict of Interest

The authors declare that the research was conducted in the absence of any commercial or financial relationships that could be construed as a potential conflict of interest.

## Publisher's Note

All claims expressed in this article are solely those of the authors and do not necessarily represent those of their affiliated organizations, or those of the publisher, the editors and the reviewers. Any product that may be evaluated in this article, or claim that may be made by its manufacturer, is not guaranteed or endorsed by the publisher.

## References

[1] Shao C Sullivan JP Girard L Augustyn A Yenerall P Rodriguez-Canales J. Essential role of aldehyde dehydrogenase 1A3 for the maintenance of non-small cell lung cancer stem cells is associated with the STAT3 pathway. Clin Cancer Res. (2014) 20:4154–66. 10.1158/1078-0432.CCR-13-3292 24907115 PMC4438754

[2] Chen CH Ferreira JC Gross ER Mochly-Rosen D. Targeting aldehyde dehydrogenase 2: new therapeutic opportunities. Physiol Rev. (2014) 94:1–34. 10.1152/physrev.00017.2013 24382882 PMC3929114

[3] Wang LS Wu ZX. ALDH2 and cancer therapy. Adv Exp Med Biol. (2019) 1193:221–28. 10.1007/978-981-13-6260-6_13 31368107

[4] Chang JS Hsiao JR Chen CH. ALDH2 polymorphism and alcohol-related cancers in Asians: a public health perspective. J Biomed Sci. (2017) 24:19. 10.1186/s12929-017-0327-y 28253921 PMC5335829

[5] Chen X Legrand AJ Cunniffe S Hume S Poletto M Vaz B. Interplay between base excision repair protein XRCC1 and ALDH2 predicts overall survival in lung and liver cancer patients. Cell Oncol (Dordr). (2018) 41:527–39. 10.1007/s13402-018-0390-8 30088263 PMC6153960

[6] Andrew AS Gui J Hu T Wyszynski A Marsit CJ Kelsey KT. Genetic polymorphisms modify bladder cancer recurrence and survival in a USA population-based prognostic study. BJU Int. (2015) 115:238–47. 10.1111/bju.12641 24666523 PMC4533837

[7] Hou G Chen L Liu G Li L Yang Y Yan HX. Aldehyde dehydrogenase-2 (ALDH2) opposes hepatocellular carcinoma progression by regulating AMP-activated protein kinase signaling in mice. Hepatology. (2017) 65:1628–44. 10.1002/hep.29006 28027570

[8] Muzio G Maggiora M Paiuzzi E Oraldi M Canuto RA. Aldehyde dehydrogenases and cell proliferation. Free Radic Biol Med. (2012) 52:735–46. 10.1016/j.freeradbiomed.2011.11.033 22206977

[9] Moreb JS Ucar D Han S Amory JK Goldstein AS Ostmark B. The enzymatic activity of human aldehyde dehydrogenases 1A2 and 2 (ALDH1A2 and ALDH2) is detected by Aldefluor, inhibited by diethylaminobenzaldehyde and has significant effects on cell proliferation and drug resistance. Chem Biol Interact. (2012) 195:52–60. 10.1016/j.cbi.2011.10.007 22079344 PMC3350780

[10] Kim J Chen CH Yang J Mochly-Rosen D. Aldehyde dehydrogenase 2^*^2 knock-in mice show increased reactive oxygen species production in response to cisplatin treatment. J Biomed Sci. (2017) 24:33. 10.1186/s12929-017-0338-8 28532411 PMC5439151

[11] Wang NN Wang LH Li Y Fu SY Xue X Jia LN. Targeting ALDH2 with disulfiram/copper reverses the resistance of cancer cells to microtubule inhibitors. Exp Cell Res. (2018) 362:72–82. 10.1016/j.yexcr.2017.11.004 29155365

[12] Liu P Brown S Goktug T Channathodiyil P Kannappan V Hugnot JP. Cytotoxic effect of disulfiram/copper on human glioblastoma cell lines and ALDH-positive cancer-stem-like cells. Br J Cancer. (2012) 107:1488–97. 10.1038/bjc.2012.442 23033007 PMC3493777

[13] Hellmann MD Callahan MK Awad MM Calvo E Ascierto PA Atmaca A. Tumor mutational burden and efficacy of nivolumab monotherapy and in combination with ipilimumab in small-cell lung cancer. Cancer Cell. (2019) 35:329. 10.1016/j.ccell.2019.01.011 30753829

[14] Lauss M Donia M Harbst K Andersen R Mitra S Rosengren F. Mutational and putative neoantigen load predict clinical benefit of adoptive T cell therapy in melanoma. Nat Commun. (2017) 8:1738. 10.1038/s41467-017-01460-0 29170503 PMC5701046

[15] Hugo W Zaretsky JM Sun L Song C Moreno BH Hu-Lieskovan S. Genomic and transcriptomic features of response to Anti-PD-1 therapy in metastatic melanoma. Cell. (2016) 165:35–44. 10.1016/j.cell.2016.02.065 26997480 PMC4808437

[16] Riaz N Havel JJ Makarov V Desrichard A Urba WJ Sims JS. Tumor and microenvironment evolution during immunotherapy with nivolumab. Cell. (2017) 171:934–49 e16. 10.1016/j.cell.2017.09.028 29033130 PMC5685550

[17] Geeleher P Zhang Z Wang F Gruener RF Nath A Morrison G. Discovering novel pharmacogenomic biomarkers by imputing drug response in cancer patients from large genomics studies. Genome Res. (2017) 27:1743–51. 10.1101/gr.221077.117 28847918 PMC5630037

[18] Charoentong P Finotello F Angelova M Mayer C Efremova M Rieder D. Pan-cancer immunogenomic analyses reveal genotype-immunophenotype relationships and predictors of response to checkpoint blockade. Cell Rep. (2017) 18:248–62. 10.1016/j.celrep.2016.12.019 28052254

[19] Hodskinson MR Bolner A Sato K Kamimae-Lanning AN Rooijers K Witte M. Alcohol-derived DNA crosslinks are repaired by two distinct mechanisms. Nature. (2020) 579:603–08. 10.1038/s41586-020-2059-5 32132710 PMC7116288

