# Supplementary material for: Molecular Characterization and Clinical Relevance of ALDH2 in Human Cancers
Source: Front Med (Lausanne). 2022 Jan 13;8:832605. doi: 10.3389/fmed.2021.832605 (PMC8792945; doi:10.3389/fmed.2021.832605)
Supplement: Supplementary file 1 [file Data_Sheet_1.docx]

**Supplementary method**

**Multi-omics data of 33 cancer types in TCGA**

The results in our analysis are based upon omics datasets generated by TCGA Research Network (<http://cancergenome.nih.gov/>). We totally analyzed 33 different TCGA projects, each project represents a specific cancer type, including KIRC, kidney renal clear cell carcinoma; KIRP, kidney renal papillary cell carcinoma; KICH, kidney chromophobe; LGG, brain lower grade glioma; GBM, glioblastoma multiforme; BRCA, breast cancer; LUSC, lung squamous cell carcinoma; LUAD, lung adenocarcinoma; READ, rectum adenocarcinoma; COAD, colon adenocarcinoma; UCS, uterine carcinosarcoma; UCEC, uterine corpus endometrial carcinoma; OV, ovarian serous cystadenocarcinoma; HNSC, head and neck squamous carcinoma; THCA, thyroid carcinoma; PRAD, prostate adenocarcinoma; STAD, stomach adenocarcinoma; SKCM, skin cutaneous melanoma; BLCA, bladder urothelial carcinoma; LIHC, liver hepatocellular carcinoma; CESC, cervical squamous cell carcinoma and endocervical adenocarcinoma; ACC, adrenocortical carcinoma; PCPG, pheochromocytoma and paraganglioma; SARC, sarcoma; LAML, acute myeloid leukemia; PAAD, pancreatic adenocarcinoma; ESCA, esophageal carcinoma; TGCT, testicular germ cell tumors; THYM, thymoma; MESO, mesothelioma; UVM, uveal melanoma; DLBC, lymphoid neoplasm diffuse large b-cell lymphoma; CHOL, cholangiocarcinoma. We downloaded the Fragments Per Kilobase of transcript per Million mapped reads (FPKM)-based gene expression for 33 types of cancer. There were no normal samples in the mRNA expression data of ACC, DLBC, LAML, LGG, MESO, OV, TGCT, UCS, and UVM.

For the CNV data, genes with focal CNV values smaller than -0.3 are categorized as a "loss" (-1), genes with focal CNV values larger than 0.3 are categorized as a "gain" (+1), and genes with focal CNV values between and including -0.3 and 0.3 are categorized as "neutral" (0).

**Immunotherapeutic cohorts**

Three independent cohorts containing immunotherapy information and expression data were collected from Gene Expression Omnibus (GEO, <http://www.ncbi.nlm.nih.gov/geo>)(1) GSE100797: melanoma patients treated with adoptive T cell therapy (ACT); (2) GSE78220: melanoma patients treated with anti-PD-1; GSE91061: melanoma patients treated with anti-PD-1. According to the RECIST v1.1 criterion, patients with complete response (CR) or partial response (PR) and patients with stable disease (SD) or progressive disease (PD) were deemed as immunotherapy responders and nonresponders, respectively, and patients who were not evaluable (NE) were removed. Ultimately, we determined 21 patients (8 responders and 13 nonresponders) in GSE100797, 28 patients (15 responders and 13 nonresponders) in GSE78220, as well as 49 patients (10 responders and 39 nonresponders) in GSE91061.

**Human cancer specimens and clinical information**

A total of 60 cancer tissues and matched adjacent nontumor tissues were enrolled from The First Affiliated Hospital of Zhengzhou University, including 5 pairs of pancreatic cancers, 10 pairs of paired liver cancers, 5 pairs of bile duct cancers, and 40 pairs of colorectal cancers. None of patients received any preoperative chemotherapy or radiotherapy. Written informed consent was obtained from all patients. The inclusion criteria were as follows: no preoperative chemotherapy, radiotherapy, or targeted therapy; no other types of tumors; no autoimmune diseases. The specimens obtained during surgery were immediately snap frozen in liquid nitrogen and stored at -80°C until RNA extraction. Clinical stage of the specimens was based on NCCN (2019) guidelines. The clinical characteristics of patients included gender, clinical stage, distant metastasis status, lymph metastasis status, vessel invasion status, nerve invasion status, disease-free survival (DFS), overall survival (OS). DFS was defined as the time from tissue collection to date of relapse or death of any cause. OS was defined as the time from tissue collection to date of death of any cause. The details of baseline information please refer to Table S1.


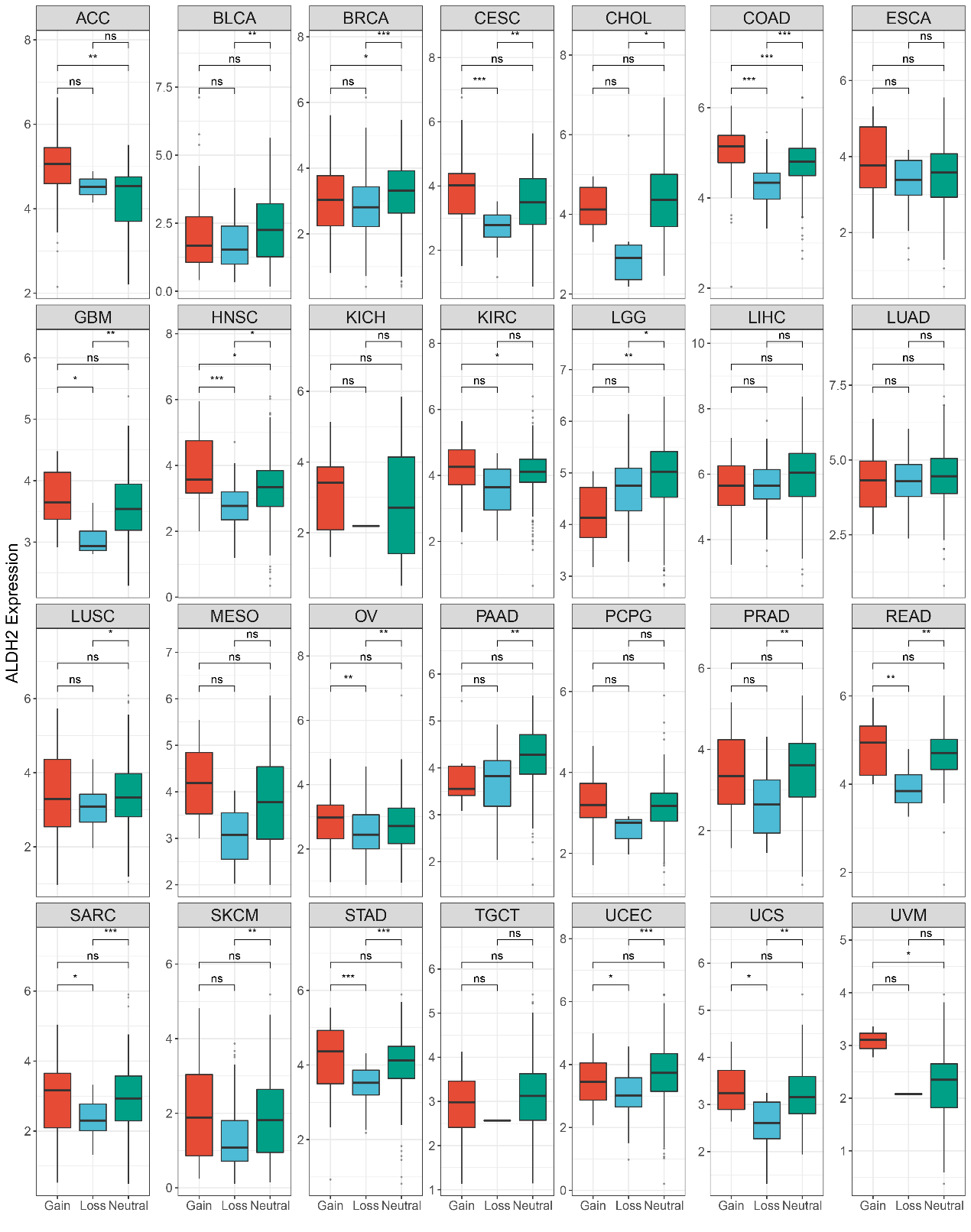


**Figure S1**. The distribution of ALDH2 expression among different CNV status groups. ns, *P* ≥0.05; *, *P* < 0.05; **, *P* < 0.01; ***, *P* < 0.001.


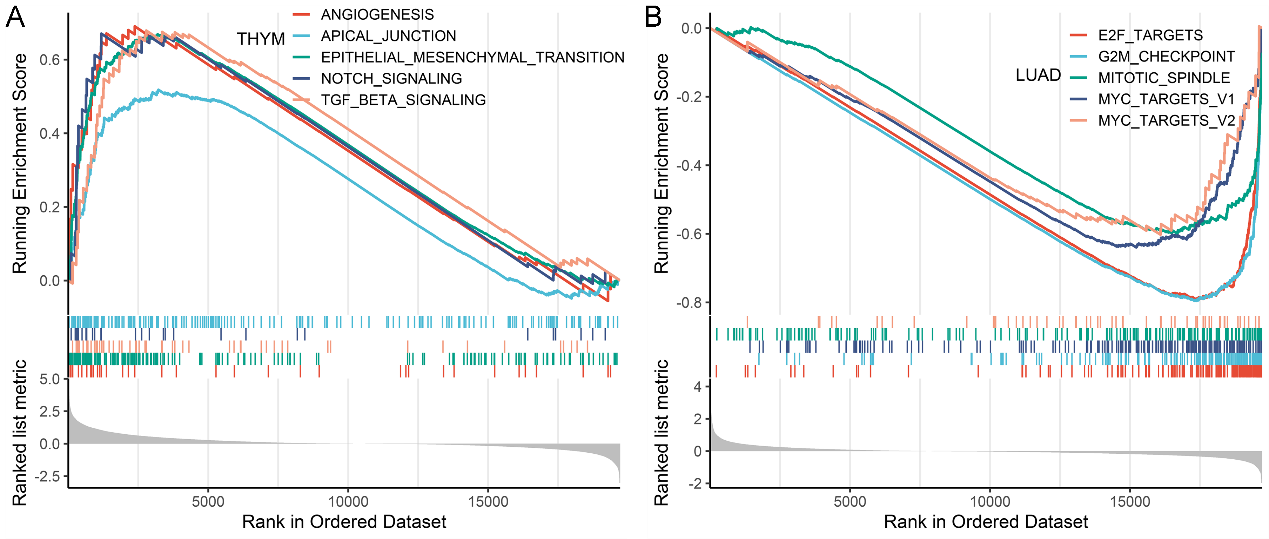


**Figure S2**. GSEA results of THYM and LUAD. The top five pathways with FDR <0.05 and NES >0 was retrieved form GSEA results of THYM (**A**) and LUAD (**B**).

**Table S1**. The details of baseline information in our cohort.

| **Total (%)** | 60 (100) |
| --- | --- |
| **Cancer (%)** |  |
| Pancreatic cancer | 5 (8.3) |
| Liver cancer | 10 (16.7) |
| Cholangiocarcinoma | 5 (8.3) |
| Colorectal cancer | 40 (66.7) |
| **Gender (%)** |  |
| Female | 39 (65.0) |
| Male | 21 (35.0) |
| **AJCC stage (%)** |  |
| Stage I | 12 (20.0) |
| Stage II | 20 (33.3) |
| Stage III | 21 (35.0) |
| Stage IV | 7 (11.7) |
| **Distant metastasis (%)** |  |
| Yes | 20 (33.3) |
| No | 40 (66.7) |
| **Lymph metastasis (%)** |  |
| Yes | 26 (43.3) |
| No | 34 (56.7) |
| **Vessel invasion (%)** |  |
| Yes | 25 (41.7) |
| No | 35 (58.3) |
| **Nerve invasion (%)** |  |
| Yes | 40 (66.7) |
| No | 20 (33.3) |
| **Disease-free status (%)** |  |
| Yes | 34 (56.7) |
| No | 26 (43.3) |
| **Survival status (%)** |  |
| Alive | 40 (66.7) |
| Dead | 20 (33.3) |
